# THBS2 Marks Disease-Associated Chondrocyte States in Knee Osteoarthritis Cartilage: An Integrative Transcriptomic and Genetic Study

**DOI:** 10.3390/genes17101299

**Published:** 2026-10-18

**Authors:** Gang Hong, Baodong Wang, Le Zhang, Xiaochuan Kong, Jiling Zhang, Tianyi Wang, Lei Zang

**Affiliations:** 1 Department of Orthopedics, Beijing Chao-Yang Hospital, Capital Medical University, 5 JingYuan Road, Shijingshan District, Beijing 100043, China; 2 Department of Clinical Laboratory, Beijing Shunyi District Hospital, No. 3 Guangming South Street, Shunyi District, Beijing 101300, China

**Keywords:** knee osteoarthritis, THBS2, extracellular matrix, focal adhesion, single-cell RNA sequencing, Mendelian randomization, pseudotime analysis, cell–cell communication

## Abstract

**Background/Objectives:** Knee osteoarthritis (KOA) is a major cause of disability, yet the molecular links between extracellular matrix (ECM) remodeling, focal adhesion signaling, and pathological chondrocyte state transitions remain incompletely defined. We aimed to identify focal adhesion-related molecules associated with KOA and characterize their genetic, tissue-level, and single-cell features. **Methods:** Global Burden of Disease (GBD) 2021 data were used only to provide epidemiologic context for KOA-related disability-adjusted life years (DALYs). Bulk transcriptomic datasets (GSE114007 and GSE169077) were analyzed for differential expression, pathway enrichment, and focal adhesion-related genes. Two-sample Mendelian randomization (MR) integrating blood-derived cis-eQTL summary statistics from eQTLGen with KOA GWAS data was used for exploratory genetic prioritization; these eQTLs were not used as a surrogate measure of cartilage THBS2 expression. THBS2 was further evaluated by expression validation, gene set enrichment, immune-related ssGSEA signature analysis, single-cell RNA sequencing (GSE255460), Slingshot pseudotime analysis, and CellChat. **Results:** As epidemiologic context, global KOA related DALYs increased from 5.17 million in 1990 to 12.12 million in 2021. Bulk analysis identified 1584 differentially expressed genes (DEGs) and 32 focal adhesion-related candidates. Blood-derived cis-eQTL-based MR showed that genetically instrumented higher THBS2 expression was associated with lower KOA risk (OR = 0.718, 95% CI:0.570–0.905, *p* = 0.00497), whereas bulk transcriptomic analyses showed higher tissue-level THBS2 expression in KOA cartilage; this bulk difference may reflect both cell-composition changes and cell-intrinsic regulation. Single-cell analysis localized THBS2 mainly to fibrochondrocytes and osteogenic chondrocytes; At the pooled-cell level, THBS2-detectable cells were more frequent in OA than control cartilage (19.45% vs. 1.44%). Pooled CellChat analysis suggested differences in several communication pathways, including higher COLLAGEN, FN1, MIF, LAMININ, and SPP1 pathway scores but a lower THBS pathway score in OA. **Conclusions:** THBS2 is a genetically prioritized, disease-associated matricellular molecule linked to ECM remodeling and selected disease-associated chondrocyte states in KOA. The CellChat findings describe parallel, broader communication remodeling in the cartilage microenvironment.

## 1. Introduction

Knee osteoarthritis (KOA) is one of the most common and disabling subtypes of osteoarthritis and has become an increasingly important public health concern in the context of global population aging. According to the Global Burden of Disease (GBD) study, approximately 595 million people worldwide were afflicted with osteoarthritis in 2020, accounting for 7.6% of the global population and representing a 132.2% increase compared with 1990 [1]. GBD 2021 estimated that the global number of prevalent KOA cases reached approximately 374.74 million in 2021, with approximately 30.85 million incident cases annually. Women, individuals older than 50 years, and populations in regions with a higher sociodemographic index (SDI) showed a greater disease burden [2]. These data indicate that KOA is not only a common chronic degenerative disease, but also a disabling condition that places long-term demands on healthcare resources and disrupts quality of life and work capacity; however, current KOA management primarily focuses on symptom control, including exercise intervention, weight management, analgesic therapy, anti-inflammatory treatment, and joint replacement at the end stage, whereas disease-modifying therapeutic strategies that effectively slow or reverse structural cartilage degeneration remain unavailable [3,4,5]. Therefore, identifying key molecules and pathological cellular states that drive KOA progression is important for early intervention and targeted therapy.

Traditionally, KOA has been considered age-related “cartilage wear and tear.” In recent years, however, it has become evident that it is a complex whole-joint disease involving articular cartilage, subchondral bone, synovium, meniscus, ligaments, and the local immune microenvironment [3,4,6,7]. Of these components, articular cartilage degeneration is a key pathological feature of KOA. Normal articular cartilage relies on a highly organized extracellular matrix (ECM) to maintain low-friction movement, compressive resistance, and load-bearing capacity. Chondrocytes are the predominant resident cells in articular cartilage. They maintain ECM homeostasis by regulating the synthesis and degradation of collagens, proteoglycans, and other matrix components. Abnormal mechanical loading, inflammatory mediators, oxidative stress, and metabolic dysregulation may disturb this balance. Under these conditions, chondrocytes may acquire inflammatory-like, hypertrophic-like, fibrotic-like, osteogenic-like, or repair-associated transcriptional features. These changes are accompanied by reduced ECM synthesis, enhanced matrix degradation, and structural cartilage damage [8,9]. Notably, ECM degradation is not merely a consequence of cartilage degeneration, as it may also promote disease progression by releasing matrix-derived fragments, altering local mechanical properties, and activating the inflammatory response. Chronic low-grade inflammation in OA interacts with matrix degradation and innate immune activation, thereby amplifying joint microenvironmental imbalance [10]. Therefore, identifying the key molecules involved in chondrocyte ECM interaction networks may clarify the mechanisms underlying the transition from tissue homeostatic imbalance to irreversible cartilage degeneration during KOA.

The integrin–focal adhesion axis plays an important role in how chondrocytes sense changes in ECM structure and the mechanical microenvironment. Integrins are transmembrane receptors that connect the ECM to the cytoskeleton. After binding to collagen, fibronectin, laminin, osteopontin, and other matrix molecules, integrins transmit extracellular mechanical and biochemical signals into cells [11,12]. Focal adhesions are multiprotein complexes consisting of integrins, focal adhesion kinase (FAK), paxillin, vinculin, and cytoskeletal adaptor proteins. They not only participate in cell adhesion and the maintenance of cell morphology, but also regulate chondrocyte survival, migration, mechanotransduction, inflammatory responses, and matrix metabolism. Integrin expression profiles and cell–matrix adhesion states are altered in OA chondrocytes, and aberrant integrin-related signaling may contribute to chondrocyte hypertrophy, ECM remodeling, and pathological changes at the osteochondral interface [11,12]. Moreover, mechanical injury can trigger mechanosensitive inflammatory signaling, whereas abnormal mechanical stress may affect chondrocyte metabolism and cartilage homeostasis through αV integrin-mediated activation of TGFβ signaling [13,14]. Nevertheless, current studies on focal adhesion-related genes in KOA have primarily focused on a limited number of candidate molecules or individual pathways. Systematic examinations integrating disease burden, tissue-level transcriptomics, genetic causal evidence, and single-cell state localization remain insufficient.

Bulk transcriptomic analysis can identify differentially expressed genes and enriched pathways at the tissue level in KOA; however, these results often represent averaged signals from multiple cell types and cellular states, making it difficult to determine whether altered expression reflects potential drivers of disease development or secondary responses to tissue injury. Moreover, differential expression analysis cannot effectively distinguish causal associations from concomitant changes alone. Mendelian randomization (MR), which uses genetic variants as instrumental variables, can reduce the effect of confounding factors and reverse causation, thereby providing genetic evidence for associations between candidate molecules and disease risk [15]. In particular, gene expression-based MR analysis using cis-expression quantitative trait loci (cis-eQTLs) can integrate genetically regulated gene expression with GWAS data from complex diseases and thereby prioritize candidate genes. The eQTLGen resource was included because it provides large-scale cis-eQTL summary statistics that enable genetic prioritization across many candidate genes; however, these eQTLs are derived primarily from blood [16]. Accordingly, the MR analysis was used to evaluate inherited, genetically proxied expression propensity in the eQTL source tissue rather than to measure or substitute for THBS2 expression in articular cartilage. Tissue specificity therefore limits direct biological extrapolation of the MR estimate to cartilage. Integrating KOA cartilage differential expression, focal adhesion-related gene sets, and eQTL-MR analysis may therefore provide complementary tissue-level and genetic evidence while addressing distinct analytical questions.

Thrombospondin-2 (THBS2) is a secreted matricellular protein and a member of the thrombospondin family. It is primarily localized in the extracellular microenvironment and participates in cell–matrix interactions, collagen fibrillogenesis, ECM assembly, angiogenesis regulation, and modulation of matrix metalloproteinase levels [17,18]. Animals deficient in TSP2 exhibit disorganized collagen fibrillogenesis, increased vascular density, and abnormal cell adhesion, which further highlights the importance of THBS2 in collagen tissue architecture and cell–matrix interactions [19]. Studies in animal and tissue repair models also suggest that THBS2 deficiency affects the relative proportion of cartilage and bone formation during fracture healing and alters the tissue repair process [20]. From a biological perspective, THBS2 is closely associated with ECM remodeling, fibrotic-like responses, altered chondrocyte adhesion, and failed tissue repair in KOA; however, the stability of THBS2 expression in KOA cartilage, its genetic causal evidence, functional pathway context, and specific cellular source have not yet been elucidated. In particular, because of the marked cellular heterogeneity of KOA cartilage, it remains unclear which chondrocyte states contribute to THBS2 expression, whether THBS2 expression changes during chondrocyte state transitions, and whether THBS2-enriched cells reside in a pathological microenvironment characterized by active ECM-related intercellular communication.

Single-cell RNA sequencing is a powerful tool for dissecting chondrocyte heterogeneity in KOA cartilage. Ji et al. identified multiple molecularly defined chondrocyte subpopulations in human OA cartilage using single-cell transcriptomic data. They proposed that distinct chondrocyte states reflect functional differentiation and pathological transitions during OA progression [21]. Single-cell studies further demonstrated that OA cartilage does not consist of a single population of “degenerative chondrocytes,” but instead contains multiple disease-associated states, including inflammatory chondrocytes, hypertrophic-like chondrocytes, fibrocartilage-like chondrocytes, repair-associated chondrocytes, and osteogenic-like chondrocytes [22,23]. These results suggest that the functional interpretation of candidate genes should not remain limited to tissue-averaged expression levels, but should be further localized to specific cellular states and pathological microenvironments. Pseudotime analysis may be used to infer continuous transcriptional changes among cellular states [24], whereas ligand–receptor inference methods, such as CellChat, enable the analysis of signaling inputs, outputs, and pathway remodeling among different cell subpopulations involved in intercellular communication [25]. Therefore, integrating single-cell localization, pseudotime trajectory analysis, and cell–cell communication analysis with bulk transcriptomic and MR results may advance studies of “candidate genes” to “key cellular states” and “pathological communication networks.”

Based on this rationale, we established an integrative molecular framework to examine the pathological significance of focal adhesion-related molecules in KOA. GBD 2021 data were retained only as a concise epidemiologic context to illustrate the clinical burden of KOA. Bulk transcriptomic data from KOA cartilage were analyzed to identify differentially expressed genes, which were intersected with a focal adhesion-related gene set to obtain KOA-associated differentially expressed focal adhesion-related genes. Next, cis-eQTL and KOA GWAS data were integrated for two-sample MR analysis to prioritize candidate genes from a genetic perspective. We focused on THBS2 and evaluated its functional context through independent cohort expression validation, gene set enrichment analysis, and immune-related ssGSEA signature analysis. Moreover, single-cell RNA sequencing and Slingshot pseudotime analysis were used to localize THBS2 across chondrocyte states, whereas CellChat was used separately to characterize broader communication patterns in the cartilage microenvironment. We hypothesize that THBS2 marks disease-associated chondrocyte states associated with ECM remodeling. A detailed flowchart of the analysis is presented in Graphical Abstract.

## 2. Materials and Methods

### 2.1. Data Sources

The data were obtained from public databases or publicly available summary statistics, including GBD 2021, bulk transcriptomic and single-cell RNA sequencing datasets from the Gene Expression Omnibus (GEO), eQTLGen blood-derived cis-eQTL summary statistics, KOA GWAS summary statistics, and GWAS Catalog annotation data. GSE114007 was used as the bulk transcriptomic discovery cohort and included 18 control and 20 KOA cartilage samples, whereas GSE169077 was used as the external validation cohort and included 5 control and 6 OA cartilage samples. The single-cell RNA sequencing dataset was GSE255460. The KOA GWAS outcome data were obtained from GWAS Catalog study GCST005813, which corresponded to the OpenGWAS/VCF identifier EBI-a-GCST005813. The group definitions and sample numbers were determined from GEO sample annotations and the processed expression matrices used in the analysis. Because the study did not involve the collection of new human samples and all datasets were publicly available and de-identified, ethical approval was not required.

### 2.2. GBD 2021 Analysis as Epidemiologic Context

GBD 2021 data were used only to provide descriptive epidemiologic context for KOA [26]. We summarized the long-term change in KOA-related disability-adjusted life years (DALYs), age- and sex-related burden patterns, and geographic heterogeneity using the publicly available GBD estimates. Bayesian age-period-cohort (BAPC) analysis was used to project future trends [27]. These analyses were intended to establish clinical context rather than to support molecular or mechanistic inference regarding THBS2.

### 2.3. Bulk Transcriptomic Data Preprocessing, Differential Expression Analysis, and Functional Enrichment Analysis

Bulk transcriptomic datasets of KOA and control cartilage tissues were obtained from the GEO database, with GSE114007 used as the discovery cohort (18 control and 20 KOA cartilage samples) and GSE169077 used as the external validation cohort (5 control and 6 OA cartilage samples). After downloading the expression matrices and corresponding sample annotation files, probe or transcript IDs were converted to standard gene symbols based on the relevant platform annotation files. If multiple probes or transcripts were mapped to the same gene, a gene-level merging strategy was used to obtain a unique expression value for each gene. Samples were classified into KOA and control groups based on the GEO sample source information and clinical annotations. Before differential expression analysis, quality control of the expression matrices was performed, including inspection of sample expression distributions, identification of outlier samples, and normalization or log2 transformation to ensure comparability of expression values among the samples.

Differential expression analysis was conducted using the limma package [28]. A design matrix was constructed with disease status as the main comparison variable, and empirical Bayes moderation was used to stabilize gene-level variance estimates. Differentially expressed genes were identified using |log2 fold change| > 1 and Benjamini–Hochberg-adjusted FDR < 0.05. Differential expression results were used for volcano plots, heatmaps, functional enrichment analysis, and screening of focal adhesion-related genes.

To characterize the major biological functions and signaling pathways associated with KOA-related differentially expressed genes, Gene Ontology (GO) and Kyoto Encyclopedia of Genes and Genomes (KEGG) enrichment analyses were conducted using clusterProfiler [29]. GO analysis focused on the biological process category, whereas KEGG analysis was used to identify significantly enriched disease-related pathways. Multiple testing was corrected using the Benjamini–Hochberg method, and FDR < 0.05 was considered statistically significant.

Focal adhesion-related genes were obtained from the KEGG human focal adhesion pathway (hsa04510), which contained 203 human genes. Differentially expressed focal adhesion-related genes (DE-FARGs) were defined as genes that met the DEG criteria (|log2 fold change| > 1 and Benjamini–Hochberg-adjusted FDR < 0.05) and were also present in the KEGG hsa04510 gene set. This intersection yielded 32 DE-FARGs: JUN, COL1A1, VEGFA, THBS2, PDGFC, COL1A2, CCND1, SHC4, THBS3, TLN2, RAC2, PAK3, FN1, TNC, PGF, LAMC3, COL6A1, COL6A3, ITGB4, COL2A1, SPP1, TNR, VEGFC, COL6A6, IGF1, COL9A1, LAMB1, TNN, SHC3, VAV1, ITGA4, and ITGB8. A protein–protein interaction (PPI) network was constructed using the STRING database [30], and the interaction characteristics of extracellular matrix, integrin, and signal transduction-related genes within the network were visualized.

### 2.4. Two-Sample Mendelian Randomization Analysis

To prioritize DE-FARGs associated with KOA risk from an exploratory genetic perspective, a two-sample MR analysis was conducted [15,31]. Genetic instrumental variables for candidate-gene expression were obtained from eQTLGen cis-eQTL summary statistics, which are derived primarily from blood [16]. The eQTLGen resource was retained because its large sample size and broad cis-eQTL coverage provide usable instruments for genetic prioritization of multiple candidate genes. In this manuscript, “genetically instrumented” or “genetically predicted” expression refers to the expression component proxied by inherited cis-eQTL variants in the eQTL source tissue; it does not refer to directly measured THBS2 expression in blood or cartilage and should not be interpreted as a surrogate for cartilage expression. Cis-eQTLs with an FDR < 0.05 were used as the exposure data source, and cis-eQTLs reaching genome-wide significance (*p* < 5 × 10^−8^) were retained for instrumental variable selection. KOA outcome data was derived from individuals of European ancestry in the UK Biobank and consisted of 4462 KOA cases and 17,885 controls, with a total sample size of 22,347. Exposure and outcome datasets were harmonized at the SNP level, and genetic variants with inconsistent alleles, ambiguous strand orientation, or unmatched information were removed. To minimize the effect of linkage disequilibrium (LD) on MR estimates, LD clumping was conducted using PLINK v1.9 with the 1000 Genomes Project European reference panel. The LD threshold, physical window size, and clumping parameters used in the analysis were r^2^ < 0.001 within a 10,000-kb window. After retaining independent genetic instruments, F statistics were calculated to evaluate instrument strength, whereas weak instruments with F ≤ 10 were excluded. For candidate genes with multiple genetic instruments, the inverse-variance-weighted (IVW) fixed-effect model was used as the primary MR method. The MR results are presented as odds ratios (ORs), 95% CIs, and *p* values, with FDR correction applied to control for multiple testing. For genes with sufficient instrumental variables, additional sensitivity analyses were conducted, including MR-Egger regression, weighted median analysis, Cochran’s Q test for heterogeneity, MR-Egger intercept test for horizontal pleiotropy, leave-one-out analysis, and MR-PRESSO analysis [32], to assess the robustness of the MR results and the potential effect of horizontal pleiotropy. Because the instruments were blood-derived, the resulting MR estimates were interpreted as genetic prioritization rather than as direct evidence of cartilage-intrinsic THBS2 expression effects. No colocalization analysis was performed in the present study. Therefore, the cis-eQTL MR analysis cannot determine whether the THBS2 expression association and the KOA GWAS association are driven by the same causal variant, or by distinct variants that are correlated through linkage disequilibrium. The MR findings were consequently treated as exploratory genetic prioritization rather than evidence of a shared causal variant.

### 2.5. THBS2-Related GSEA, and Immune-Related ssGSEA Signature Analysis

To determine the molecular functions associated with THBS2, correlations between THBS2 expression and the whole-gene expression profile were calculated based on bulk transcriptomic data, and genes were ranked based on correlation coefficients or differential statistics. Gene set enrichment analysis (GSEA) was conducted using Hallmark gene sets from the Molecular Signatures Database (MSigDB) [33] to identify biological processes and signaling pathways associated with high THBS2 expression. GSEA results are presented as normalized enrichment scores (NESs), nominal *p* values, and FDR values; FDR < 0.05 was considered statistically significant. For immune-related transcriptional context, single-sample GSEA (ssGSEA) was applied to predefined immune cell- and immune function-related gene sets to generate signature scores for each bulk cartilage sample. These ssGSEA scores were treated as relative gene-set enrichment measures rather than direct estimates of immune-cell abundance or proof of the physical presence of specific immune-cell populations. This distinction is particularly important for low-cellularity cartilage, in which generic immune gene signatures may reflect shared inflammatory, antigen-presentation, or stress-response transcriptional programs. Differences in immune-related signature scores between KOA and control samples were compared, and Spearman correlation analysis was used to examine associations between THBS2 expression and these signature scores. Results are presented as correlation coefficients (r), *p* values, and FDR-adjusted values. Because the bulk cartilage datasets measure tissue-averaged expression, the observed THBS2 difference represents a composite signal across all cells present in each specimen. The bulk analysis did not adjust THBS2 expression for cell-type composition and therefore cannot distinguish increased THBS2 expression within the same cell state from a higher relative abundance of THBS2-rich cell states.

### 2.6. Single-Cell RNA Sequencing Data Processing and Cell Annotation

The KOA single-cell RNA sequencing dataset GSE255460, which consists of 19 sequencing libraries, was obtained from the GEO database. Individual sample objects were constructed using Seurat and merged for downstream analysis [34]. Before quality control, a total of 150,706 cells were obtained. The cells were filtered based on nFeature_RNA, nCount_RNA, and percent.mt. Cells were retained with nFeature_RNA between 800 and 6000, nCount_RNA between 1500 and 25,000, and percent.mt < 8%. Finally, 121,825 high-quality cells were retained for subsequent analyses.

The quality-controlled expression matrix was normalized using the LogNormalize method with a scale factor of 10,000, and 3000 highly variable genes were identified using FindVariableFeatures. ScaleData, principal component analysis, neighbor graph construction, clustering analysis, and UMAP dimensionality reduction were sequentially conducted. The number of PCs used for clustering and UMAP, clustering resolution, random seed, and marker selection parameters are presented in the Supplementary Methods. To correct for batch effects among samples, Harmony integration was conducted based on sample origin [35], followed by re-clustering and UMAP visualization in the Harmony-corrected low-dimensional space. To make the annotation procedure explicit, no single automated reference-mapping database was used as the final annotation criterion. Positive marker genes identified for each Harmony cluster were ranked and inspected, and cluster identities were assigned by integrating marker DotPlot/FeaturePlot patterns with canonical lineage markers and published OA cartilage single-cell marker definitions [21,22,23]. Representative markers used for annotation included COL2A1, ACAN, COL9A1/COL11A1, and MATN1 for matrix chondrocytes; PRG4, CRTAC1, and ABI3BP for reparative chondrocytes; COL1A1/COL1A2, COL3A1, FN1, and POSTN for fibrochondrocytes; COL10A1, MMP13, SPP1, IBSP, ALPL, and RUNX2 for hypertrophic mineralizing or osteogenic chondrocytes; IL6, CCL2, CXCL8, PTGS2, and NOS2 for inflammatory and NOS2+ inflammatory chondrocytes; HSPA1A, HSPA1B, DNAJB1, FOS, and JUN for stress-response chondrocytes; C3, TNFAIP3, NFKBIA, and BMP2 for regulatory or OA-activated chondrocyte states; PTPRC, CD14, CD68, LYZ, HLA-DRA, and CD74 for macrophages; and PECAM1, VWF, CDH5, and CLDN5 for endothelial cells. Clusters without coherent canonical marker enrichment or showing low-quality transcriptional profiles were conservatively annotated as unassigned low-quality cells.

To determine the cellular source of THBS2 in the KO cartilage microenvironment, FeaturePlot, DotPlot, and violin plots were used to visualize its expression at three levels: the UMAP embedding, cell types, and disease groups. To avoid misinterpreting low-level transcript detection as protein positivity, cells with THBS2 expression > 0 in the Seurat-normalized RNA matrix were defined as “detectable cells” rather than THBS2-protein-positive cells; this definition reflects transcript detectability only. For the composition analysis, cells from the control and OA groups were pooled to calculate the relative proportion of each cell type, and the stacked bar plots are intended for descriptive purposes only; rigorous differential abundance analysis should instead calculate cell type proportions for each sample at the donor level and compare groups using appropriate sample-level or compositional models. Given that scRNA-seq transcript detection is influenced by library size/sequencing depth, stochastic dropout, sample recovery rates, and cell-state composition, this percentage is descriptive only; more rigorous validation should employ prespecified raw count thresholds (≥ 1 and higher UMI cutoffs), assess robustness across strata of sequencing depth, and aggregate counts by donor and cell state for pseudobulk comparisons. In downstream analyses, unassigned low-quality cells were excluded from the denominators of cell states and were displayed separately as a quality-control category in the global UMAP, rather than being interpreted as cell states.

### 2.7. Slingshot Pseudotime Analysis

To explore the transcriptional state continuity among chondrocyte subpopulations, Slingshot pseudotime analysis was performed [24]. The analysis focused on chondrocyte-related subpopulations; unassigned low-quality cells and all non-target populations were excluded before constructing the input object, and a maximum of 1500 cells per cell type–group combination were downsampled. When inferring a shared transcriptional continuum, control and OA chondrocytes were included jointly, aiming to compare the relative state occupancy of the two groups within the same transcriptional state space rather than to reconstruct a temporal sequence from normal to disease. A SingleCellExperiment object was constructed using Harmony-integrated UMAP coordinates and cell type labels, with the starting cluster, lineage settings, and downsampling seed prespecified to ensure reproducibility. The starting cluster was designated operationally, and no independent lineage-tracing evidence supports its representing a true biological origin; UMAP is optimized for neighborhood-preserving visualization. Within the shared manifold, the pseudotime distributions of the two groups were compared descriptively, and THBS2 expression levels together with the proportion of detectable transcripts were summarized along the inferred continuum. Given that OA cells contribute to the geometry of the jointly inferred trajectory, relative pseudotime positions cannot independently define normal precursor states, disease-induced states, or the temporal direction of KOA progression; in future studies, a reference trajectory could be inferred from control chondrocytes to determine which OA cells could be mapped using a validated projection strategy. Pseudotime direction is anchored by the selected root state, and changing the starting cluster may reverse the ordering of cells and the “early/late” positioning of THBS2. Given that the data are cross-sectional, pseudotime reflects inferred transcriptional order rather than actual temporal progression, and the related results should be regarded as hypothesis-generating descriptive findings only.

### 2.8. Cell–Cell Communication Analysis

CellChat was used to perform cell–cell communication analysis separately in the control and osteoarthritis (OA) groups. After removing unassigned low-quality cells, the normalized expression matrix of the annotated Seurat object was used as input, and cell identities were defined based on the retained cell-state annotations. Given the focus of this study on the extracellular matrix, adhesion, and cartilage microenvironment remodeling, the analysis retained primarily ligand–receptor pathways classified as “secreted signaling” and “ECM–receptor.” For each group, a maximum of 200 cells per cell type were drawn by downsampling, with a fixed random seed and downsampling strategy applied to ensure reproducibility. The CellChat workflow included: identification of overexpressed genes and ligand–receptor interactions, computation of communication probabilities, trimming of interactions involving small cell counts, computation of pathway-level communication probabilities, integration of the communication network, and centrality analysis [25]. Differential communication strength was defined as the difference between the OA and control groups (δ strength = OA − control) to identify communication directions and key pathways inferred to be enhanced or attenuated under KOA conditions. Accordingly, group differences in network features—including total interaction counts and strengths, sender/receiver centrality, and pathway-level δ strengths—were regarded as descriptive network comparisons only, as they may be influenced by the number of contributing cells per state, sequencing depth, transcript detection levels, sample composition, and the downsampling strategy. More rigorous validation should construct donor-level communication summaries and statistically compare network features across biological samples.

### 2.9. Statistical Analysis

Statistical analyses were primarily performed using R version 4.5.3. For continuous variables that did not satisfy the assumptions required for parametric testing, comparisons between two independent groups were performed using the Wilcoxon rank-sum test. Continuous data are presented as medians and interquartile ranges where appropriate. Associations between continuous variables were assessed using Spearman correlation analysis. For analyses involving multiple comparisons, the Benjamini–Hochberg method was used to control the false discovery rate (FDR). Unless otherwise specified, a two-sided *p* < 0.05 was considered statistically significant.

## 3. Results

### 3.1. GBD 2021 Provides Epidemiologic Context for the Clinical Burden of KOA

Based on GBD 2021 data, the global number of knee osteoarthritis (KOA)-related disability-adjusted life years (DALYs) increased from 5.17 million (95% UI: 2.48–11.23 million) in 1990 to 12.12 million (95% UI: 5.83–26.24 million) in 2021, representing a 134.6% increase. During the same period, the crude DALY rate increased from 96.80 to 152.96 per 100,000 population, while the age-standardized DALY rate increased from 127.28 (95% UI: 61.31–275.72) to 137.88 (95% UI: 66.26–298.53) per 100,000 in the population, with an estimated annual percentage change (EAPC) of 0.34 (95% CI: 0.31–0.37) (Figure 1A; Supplementary Table S1). In 2021, the absolute number of DALYs peaked in the 55–59-year age group, whereas the age-specific DALY rate peaked at approximately 80–84 years; DALY rates were also higher in females than in males across most age groups (Figure 1B). Marked variation in age-standardized DALY rates was observed across sociodemographic index (SDI) groups, GBD regions, and countries or territories, with relatively higher burdens in high-middle- and high-SDI regions, as well as in high-income countries in the Asia Pacific and East Asia (Figure 1C; Supplementary Table S2). Bayesian age-period-cohort (BAPC) projections suggested that the global age-standardized DALY rate may decline slightly after 2021 but remain at a relatively high level (Figure 1D). These estimates were included solely to provide an epidemiological context for the subsequent molecular analyses.

### 3.2. Identification of Differentially Expressed Focal Adhesion-Related Genes in KOA

We next focused on the molecular analyses that directly address the study hypothesis and examined KOA-associated alterations at the bulk transcriptomic level. Comparison of gene expression profiles between KOA and control cartilage identified 1584 differentially expressed genes (DEGs). Volcano plots and heatmaps illustrate the overall distribution and expression patterns of the DEGs, respectively (Figure 2A,B). Functional enrichment analysis showed that the DEGs were primarily enriched in biological processes related to angiogenesis, extracellular matrix organization, extracellular structure organization, collagen-containing extracellular matrix, cell adhesion, and skeletal system development (Figure 2C). KEGG pathway analysis showed enrichment of pathways associated with matrix remodeling, immune–inflammatory responses, and adhesion-related signaling, including complement and coagulation cascades, protein digestion and absorption, integrin signaling, PI3K-Akt signaling, ECM–receptor interaction, cell adhesion molecule interaction, osteoclast differentiation, and HIF-1 signaling (Figure 2D). Because focal adhesion-related genes are involved in chondrocyte ECM sensing, cell adhesion, and mechanotransduction, we intersected the DEGs with 203 focal adhesion-related genes (FARGs), identifying 32 differentially expressed focal adhesion-related genes (DE-FARGs), including COL1A1, COL1A2, THBS2, SPP1, JUN, FN1, VEGFA, ITGA4, ITGB8, and COL6A1 (Figure 2E). A protein–protein interaction (PPI) network based on the DE-FARGs showed a highly connected extracellular matrix- and integrin-related module, with additional connections involving regulatory genes such as JUN, IGF1, VEGFC, and CCND1 (Figure 2F). Taken together, the bulk transcriptomic results indicate that DEGs in KOA cartilage are associated with extracellular matrix remodeling, cell adhesion, inflammatory responses, and abnormal mechanotransduction.

### 3.3. MR Prioritizes Focal Adhesion-Related Candidate Genes Associated with KOA

To genetically prioritize focal adhesion-related DEGs associated with KOA, we performed two-sample MR analyses using blood-derived cis-eQTLs as exposure instruments and KOA GWAS data as the outcome. Among candidate genes with available genetic instruments, THBS2, SPP1, and JUN showed nominal evidence of association with KOA risk (Table 1). Genetically instrumented higher THBS2 expression was associated with a lower MR-estimated risk of KOA (IVW fixed-effects model: OR = 0.718, 95% CI:0.570–0.905, *p* = 0.00497), and this association remained significant after FDR correction. SPP1 (OR = 0.919, 95% CI:0.852–0.991, *p* = 0.0278) and JUN (OR = 1.177, 95% CI:1.005–1.379, *p* = 0.0434) reached nominal significance but did not remain significant after FDR correction. Sensitivity analyses did not detect substantial heterogeneity or horizontal pleiotropy among genes with multiple SNP instruments, and the corresponding forest plots, funnel plots, scatter plots, and leave-one-out analyses are shown in Supplementary Figures S1–S4. Because the exposure instruments were derived from blood and colocalization was not assessed, these MR findings were interpreted as exploratory genetic prioritization rather than evidence of a cartilage-specific causal effect. Overall, THBS2 was prioritized for subsequent transcriptomic and single-cell analyses.

### 3.4. THBS2 Is Stably Upregulated in KOA and Associated with Extracellular Matrix Remodeling and Immune-Related Transcriptional Signatures

Expression analysis showed that THBS2 was significantly upregulated in KOA cartilage in the discovery cohort GSE114007 (median expression: 10.10 vs. 7.68, *p* < 0.001; Figure 3A), and this finding was replicated in the independent validation cohort GSE169077 (10.56 vs. 8.22, *p* = 0.00866; Figure 3B). Hallmark GSEA showed that high THBS2 expression was associated with enrichment of epithelial–mesenchymal transition (EMT), apical junction, complement, protein secretion, and coagulation pathways in GSE114007 (Figure 3C; Supplementary Table S3). In GSE169077, EMT, protein secretion, and apical junction remained significantly enriched (Figure 3D; Supplementary Table S4).

ssGSEA further identified differences in multiple immune-related transcriptional signatures between KOA and control cartilage. Immature dendritic cell-, macrophage-, activated dendritic cell-, regulatory T cell-, T follicular helper cell-, and APC co-stimulation-related signature scores were higher in KOA, whereas MHC class I-, type 17 T helper cell-, inflammation-promoting, eosinophil-, activated B cell-, and activated CD8 T cell-related scores were lower (Figure 3E). THBS2 expression was positively correlated with macrophage-related (r = 0.623, FDR = 3.18 × 10^−4^), immature dendritic cell-related (r = 0.645, FDR = 1.84 × 10^−4^), T follicular helper cell-related (r = 0.582, FDR = 8.97 × 10^−4^), regulatory T cell-related (r = 0.562, FDR = 0.00142), and APC co-stimulation (r = 0.433, FDR = 0.020) signature scores (Figure 3F; Supplementary Table S5). These ssGSEA results were interpreted as immune-related transcriptional signatures rather than direct estimates of immune-cell abundance.

### 3.5. Single-Cell Atlas Reveals That THBS2 Is Primarily Localized to Fibrochondrocytes and Osteogenic Chondrocytes

After quality control, normalization, highly variable gene selection, dimensionality reduction, Harmony-based batch correction, clustering, and manual annotation, 121,825 cells were retained for downstream single-cell analysis. A total of 13 cell states or cell types were identified, including reparative chondrocytes, matrix chondrocytes, inflammatory chondrocytes, fibrochondrocytes, hypertrophic mineralizing chondrocytes, stress-response chondrocytes, and regulatory-activated chondrocytes, OA-activated chondrocytes, NOS2+ inflammatory chondrocytes, osteogenic chondrocytes, macrophages, endothelial cells, and unassigned low-quality cells (Figure 4A). Canonical marker expression supported the major cell-state annotations (Figure 4B), while Harmony integration reduced sample-specific batch effects (Supplementary Figure S5A–D).

The pooled-cell composition analysis showed descriptive differences in the relative distribution of annotated cell states between Control and OA samples (Figure 4C), with corresponding differences in cell-state occupancy observed in the group-stratified UMAP (Supplementary Figure S5E). Because cell proportions were not statistically compared at the donor level, these differences were interpreted descriptively.

THBS2 showed a nonuniform distribution across the single-cell atlas and was concentrated in specific chondrocyte populations (Figure 4D). DotPlot analysis demonstrated the highest THBS2 expression intensity and detectable-cell proportion in fibrochondrocytes, followed by osteogenic chondrocytes and several other disease-associated chondrocyte states (Figure 4E). Unassigned low-quality cells were excluded from downstream composition, THBS2-specific, pseudotime, and CellChat analyses.

### 3.6. Distribution of THBS2-Detectable Cells Along the Jointly Inferred Pseudotime Continuum

Using the Harmony-corrected UMAP representation, Slingshot reconstructed a shared transcriptional continuum across jointly analyzed Control and OA chondrocytes (Figure 5A). Control and OA cells showed different occupancy patterns within this common transcriptional state space (Figure 5B; Supplementary Figure S6A). Under the predefined trajectory orientation, OA chondrocytes showed a broader pooled-cell pseudotime distribution and a lower median pseudotime than Control chondrocytes (11.29 [IQR: 6.45–14.24] vs. 12.94 [IQR: 11.46–14.16]; Figure 5C). Because these values were summarized from pooled cells without donor-level statistical testing, they were interpreted descriptively.

THBS2 transcript levels and the pooled proportion of THBS2-detectable cells were higher in OA cells and were concentrated in specific regions of the inferred continuum (Figure 5D,E). The pooled frequency of THBS2-detectable cells was 19.45% in OA and 1.44% in the Control. Because this comparison was not donor-aware and THBS2 detectability may be influenced by transcript dropout and cell-state composition, these findings were interpreted as exploratory descriptive associations between THBS2 and OA-enriched transcriptional states rather than evidence of temporal disease progression.

### 3.7. CellChat Analysis Reveals Altered Intercellular Communication and ECM-Related Signaling Patterns in KOA

CellChat analysis was performed separately on pooled Control and OA cells to characterize inferred intercellular communication patterns in the KOA cartilage microenvironment. The pooled OA network showed a larger number of inferred interactions than the pooled Control network (3078 vs. 1831) and a higher aggregate communication-strength metric (69.2 vs. 48.3; Figure 6A). Differential network visualization further revealed altered sender–receiver communication patterns between the two groups (Figure 6B; Supplementary Figure S6B).

Macrophage-directed incoming signaling, together with outgoing signaling from osteogenic chondrocytes, fibrochondrocytes, and regulatory activated chondrocytes, was higher in the pooled OA network (Figure 6C,D). At the pathway level, positive OA-minus-Control delta-strength values were observed for COLLAGEN (18.18), FN1 (2.91), MIF (2.66), LAMININ (0.57), SPP1 (0.55), and ANNEXIN, whereas THBS, CypA, and VISFATIN showed negative contrasts (Figure 6E). Because these comparisons were based on pooled-cell networks rather than donor-level statistical analyses, they were interpreted as exploratory differences in the inferred communication structure. Notably, the THBS pathway showed a negative contrast in OA, indicating that the broader communication remodeling identified by CellChat should not be attributed specifically to THBS2.

## 4. Discussion

In this study, we characterized THBS2-associated focal adhesion–extracellular matrix (ECM) remodeling features in knee osteoarthritis (KOA) by integrating bulk transcriptomics, focal adhesion-related gene screening, cis-eQTL-based MR, external cohort validation, single-cell transcriptomics, pseudotime analysis, and cell–cell communication analysis. First, the analysis of GBD 2021 data confirmed that the disease burden of KOA continues to increase globally and exhibits marked heterogeneity by age, sex, and geographic region, and highlights the need to identify the molecules and potential therapeutic targets involved in disease progression. At the transcriptomic level, DEGs in KOA cartilage were significantly enriched in ECM organization, cell adhesion, integrin signaling, PI3K-Akt signaling, and ECM–receptor interaction pathways. This suggests that cartilage degeneration is not merely the consequence of matrix degradation, but rather a dynamic pathological process involving cell–matrix interactions, mechanotransduction, inflammatory responses, and cellular state alterations. By integrating focal adhesion-related gene sets with MR analysis, THBS2 was prioritized as a key candidate molecule supported by stable expression changes, genetic association evidence, and single-cell localization. Single-cell analysis indicated that THBS2 was primarily enriched in fibrochondrocytes and osteogenic chondrocytes, with more pronounced expression in OA-derived cells. Pseudotime analysis placed THBS2-detectable cells within specific regions of a jointly inferred transcriptional continuum; these pooled-cell patterns are descriptive and do not establish temporal progression or a statistically validated donor-level group difference. Pooled CellChat analysis suggested differences in inferred intercellular communication structure and several ECM-related pathway scores between OA and Control. These observations are analytically parallel to, rather than mechanistically downstream of, the THBS2 findings. Because the THBS pathway itself decreased in the pooled OA network, the current data do not support THBS2 as a cause or mediator of the broader communication remodeling. Taken together, these results suggest that THBS2 is associated with ECM remodeling, abnormal cell adhesion, and disease-associated chondrocyte states in KOA cartilage; the cross-sectional data do not establish directional state progression.

In the bulk transcriptomic analysis, DEGs in KOA cartilage were primarily enriched in ECM organization, collagen-containing extracellular matrix, cell adhesion, integrin signaling, ECM–receptor interaction, and PI3K-Akt pathways. These results are consistent with the core pathological features of KOA cartilage, such as the disruption of ECM homeostasis, abnormalities in the collagen network, altered chondrocyte adhesion, and impaired mechanical sensing. Normal articular cartilage relies on a highly organized ECM primarily consisting of type II collagen and proteoglycans to maintain compressive resistance and low-friction movement; however, during KOA progression, chondrocytes exposed to abnormal mechanical loading, inflammatory stimuli, and oxidative stress may undergo phenotypic drift, resulting in insufficient matrix synthesis and enhanced matrix degradation [8]. The integrin–focal adhesion axis is a key hub through which chondrocytes sense ECM alterations and mechanical cues. Through its components, such as FAK, paxillin, and vinculin, extracellular signals can be transmitted into cells to regulate chondrocyte survival, migration, mechanotransduction, inflammatory responses, and matrix metabolism [11,12]. Integrin-mediated mechanotransduction regulates early chondrocyte responses to mechanical stimulation through intracellular signaling cascades that influence gene expression and tissue remodeling [36]. In addition, fibronectin fragments can activate FAK and MAPK signaling in chondrocytes, suggesting that ECM degradation fragments may act as pro-degenerative stimuli contributing to abnormal chondrocyte activation [37]. Therefore, the 32 differentially expressed focal adhesion-related genes identified in the present study should not be interpreted as isolated expression changes, but rather as representing systematic adaptation and dysregulation of KOA chondrocytes within an abnormal matrix microenvironment. The PPI network also revealed that COL1A1, COL1A2, FN1, SPP1, THBS2, and integrin-related genes form a closely connected interaction module, which supports the central role of the ECM–integrin–FAK network in KOA cartilage remodeling.

The MR analysis genetically prioritized THBS2 among the focal adhesion-related candidates; however, the direction of this association must be interpreted cautiously. The eQTLGen instruments used in the present analysis are derived primarily from blood rather than articular cartilage or synovium. Therefore, the MR estimate (OR = 0.718) reflects the association between genetically instrumented THBS2 expression in the eQTL source context and KOA susceptibility and does not demonstrate that increasing THBS2 expression within cartilage would protect against KOA. In contrast, the bulk and single-cell transcriptomic analyses directly measured disease-state expression in cartilage, where THBS2 was upregulated. These observations address different biological and analytical questions and should not be interpreted as two opposing estimates of a single cartilage-intrinsic effect. Explanations such as a compensatory repair response, a secondary pathogenic response, tissue-specific regulation of THBS2, or disease-associated changes in cell composition remain hypotheses only. The present data cannot determine whether cartilage THBS2 is protective, pathogenic, compensatory, or simply associated with tissue remodeling. We therefore interpret the MR result only as exploratory genetic prioritization. Cartilage- or synovium-specific eQTL analyses, ideally combined with colocalization and direct functional perturbation studies, will be required to determine the direction and tissue-specific biological role of THBS2 in KOA. In addition, because colocalization was not performed, the present MR result cannot distinguish a shared causal variant from separate THBS2 eQTL and KOA-associated variants that are in linkage disequilibrium [38]. Accordingly, we do not regard the current genetic evidence as establishing a shared causal signal. The THBS2 MR result is additionally constrained by the availability of only three SNP instruments. Consequently, MR-Egger regression, heterogeneity testing, pleiotropy testing, and leave-one-out analyses are underpowered in this setting. A failure to detect heterogeneity or horizontal pleiotropy should therefore not be interpreted as demonstrating robustness of the THBS2 estimate. Tissue specificity of gene regulation is an additional source of uncertainty. A cis-eQTL that influences THBS2 expression in blood may have a weaker, absent, or even directionally different effect in articular cartilage or synovium [39]. Accordingly, the present blood-based MR estimate should not be extrapolated directly to cartilage-intrinsic THBS2 biology. We therefore interpret the MR result as a tissue-nonspecific genetic prioritization signal rather than as evidence for a cartilage-specific causal effect.

The stable upregulation of THBS2 in two independent bulk transcriptomic cohorts further supports its role as a KOA-associated candidate molecule [40]. GSEA revealed that the high THBS2 expression state was associated with EMT, apical junction, protein secretion, complement, and coagulation pathways. The Hallmark EMT pathway, in the context of cartilage tissue, should not be interpreted mechanically as canonical epithelial–mesenchymal transition. Instead, it more likely reflects enhanced mesenchymal-like features, ECM remodeling, altered cell adhesion or junction organization, and fibrotic-like responses. The ssGSEA analysis additionally identified THBS2-associated immune-related transcriptional signatures. However, because articular cartilage is a low-cellularity tissue and the analysis used generic bulk-tissue gene sets, these scores cannot reliably establish the physical presence or abundance of T follicular helper cells, regulatory T cells, dendritic cells, activated B cells, or activated CD8 T cells. They may instead capture overlapping inflammatory, antigen-presentation, or stress-response programs expressed by multiple cell types. Accordingly, we interpret these findings as part of an immune-related transcriptional context rather than as direct immune-cell infiltration estimates. The single-cell dataset provides some support for macrophage-related signals because macrophages were directly recovered, but it did not identify the full set of lymphoid and dendritic populations implied by the bulk signatures. The bulk ssGSEA and single-cell findings are therefore complementary but not quantitatively equivalent measures. The discordance between the bulk ssGSEA labels and the single-cell cell-type atlas further supports this interpretation. Because the single-cell analysis identified macrophages and endothelial cells but not the broad range of lymphoid and dendritic populations suggested by bulk ssGSEA, at least some of the bulk ‘immune’ signals likely reflect shared transcriptional programs rather than discrete immune-cell abundance. Such programs may include inflammatory signaling, antigen presentation, interferon- or stress-associated responses expressed by chondrocytes or other cells in diseased cartilage. Accordingly, cross-method agreement was considered strongest for macrophage-related signals and substantially weaker for the other immune labels. Importantly, the reproducible bulk-tissue increase in THBS2 should not be equated with cell-intrinsic upregulation. Bulk expression is a mixture of per-cell expression and tissue cellular composition, and the single-cell data indicate that THBS2 is concentrated in fibrochondrocyte and osteogenic chondrocyte states. Thus, expansion or preferential recovery of these THBS2-rich populations in OA could contribute substantially to the higher tissue-level THBS2 signal. Conversely, a within-state increase in THBS2 may also occur, but this was not established by the present donor-level analyses. We therefore interpret the bulk result as a reproducible tissue-level association whose cellular basis remains unresolved.

We found that THBS2 was not uniformly expressed across all chondrocytes but was primarily enriched in fibrochondrocytes and osteogenic chondrocytes. Previous studies have shown that OA cartilage contains multiple disease-associated chondrocyte states, including inflammatory, hypertrophic-like, fibrocartilage-like, repair-associated, and osteogenic-like chondrocytes [21,22,23]. Recent studies combining bulk and single-cell transcriptomic data have focused on molecular features of fibrocartilage chondrocytes and suggest that fibrocartilage chondrocytes are closely associated with OA cartilage degeneration and candidate biomarker identification [41]. Our results are consistent with these observations and localize THBS2 to chondrocyte states with fibrotic and osteogenic-like characteristics. Fibrochondrocytes are typically associated with type I collagen, fibronectin, and abnormal ECM deposition, features that are consistent with fibrotic-like repair responses following cartilage injury. Therefore, enrichment of THBS2 in these cell states suggests a potential association with fibrotic and osteogenic-like remodeling in KOA. The apparent differences in cell composition between Control and OA, as well as the difference in the pooled proportion of THBS2-detectable cells (19.45% vs. 1.44%), were based on pooled-cell analyses rather than donor-level statistical comparisons. These observations may therefore be influenced by unequal cell recovery, sequencing depth, dropout, and differences in cell-state composition. In particular, because THBS2 is enriched in fibrochondrocytes, the higher pooled frequency of THBS2-detectable cells in OA may partly reflect enrichment of THBS2-high cell states rather than a uniform increase in THBS2 expression within comparable cell states. Donor-level compositional analyses and cell-state-specific pseudobulk comparisons will be required for further validation. In addition, the cell-state annotations in this study were based primarily on canonical marker genes and the published literature. Highly specific OA-related labels should therefore be regarded as descriptive transcriptional states rather than definitive cell identities. Unassigned low-quality cells were retained only for quality-control visualization and were excluded from downstream THBS2, pseudotime, and CellChat analyses.

The enrichment of THBS2 in osteogenic chondrocytes is particularly noteworthy. During OA progression, a subset of chondrocytes may acquire hypertrophic and osteogenic-like differentiation features, which are accompanied by activation of molecular programs involving RUNX2, MMP13, ADAMTS5, and SPP1. Animal studies have indicated that chondrocyte-specific deletion of Runx2 attenuates DMM-induced OA-like changes and reduces the expression of matrix-degrading enzyme-related genes. This suggests that osteogenic or hypertrophic-like chondrocyte states are not merely accompanying phenomena, but may actively contribute to cartilage destruction and subchondral bone alterations [42]. Recent single-cell studies of osteochondral tissues further identified KOA-associated chondrocyte subtypes, such as Col1a1+ osteogenic chondrocytes, thereby emphasizing the connection between osteogenic transdifferentiation of chondrocytes and subchondral bone remodeling [43]. Combined with the single-cell results in the present study, the enrichment of THBS2 in osteogenic chondrocytes may reflect its involvement in abnormal chondrocyte differentiation, matrix mineralization, and pathological remodeling of the cartilage–bone interface in KOA.

The pseudotime analysis was performed jointly on Control and OA chondrocytes to characterize their distribution within a shared transcriptional state space. Accordingly, the inferred trajectory should be interpreted as transcriptional-state continuity rather than as a temporal progression from normal to pathological chondrocytes. The lower pooled pseudotime values observed in OA and the localization of THBS2-detectable cells to specific trajectory regions therefore do not indicate an “early” disease stage or directional progression. Slingshot pseudotime reflects transcriptomic similarity rather than true lineage tracing, and the present cross-sectional data cannot establish temporal evolution. In addition, the pooled-cell analysis was not validated at the donor level, and trajectory geometry may depend on the embedding space and root selection. Therefore, these findings should be regarded as exploratory associations between THBS2- and OA-enriched chondrocyte states that require donor-aware and independent validation.

CellChat analysis revealed exploratory differences in inferred intercellular communication between pooled OA and Control cartilage, including increased overall network activity and altered macrophage-directed, fibrochondrocyte/osteogenic, and ECM-related signaling patterns [44]. However, because the analysis was based on pooled cells rather than donor-specific networks, these differences should be interpreted cautiously and may be influenced by cell abundance, sequencing depth, sample composition, and downsampling. Accordingly, the observed contrasts are considered hypothesis-generating communication patterns that require donor-aware validation. At the pathway level, COLLAGEN, FN1, MIF, LAMININ, and SPP1 showed positive OA-minus-Control contrasts, whereas the THBS pathway showed a negative contrast. Therefore, the CellChat results do not support THBS2 as a specific driver of the broader communication remodeling observed in OA. Overall, these findings are better interpreted as a complementary microenvironmental context that is analytically parallel to, rather than mechanistically downstream of, the THBS2-associated observations.

Several limitations should be acknowledged. First, the MR analysis relied on blood-derived cis-eQTL instruments rather than cartilage- or synovium-specific regulatory variants, and no colocalization analysis was performed. Moreover, the THBS2 estimate was based on only three SNP instruments. Therefore, the MR findings should be interpreted as exploratory genetic prioritization rather than evidence of a cartilage-specific causal effect, and the discordance between the inverse MR association and increased THBS2 expression in KOA cartilage remains unresolved. Second, the single-cell analyses were largely based on pooled cells rather than donor-level statistical comparisons. Consequently, apparent differences in cell-state composition and THBS2-detectable cell frequencies may be influenced by unequal cell recovery, sequencing depth, dropout, and sample composition. The current data therefore cannot distinguish the contribution of altered cell composition from cell-intrinsic THBS2 upregulation. Third, the pseudotime and CellChat analyses were exploratory and based on cross-sectional, pooled-cell data. The Slingshot trajectory does not establish temporal progression from normal to pathological chondrocyte states, and the CellChat results do not demonstrate true disease-dependent signaling activity or implicate THBS2 as a causal driver of broader communication remodeling. Donor-aware, tissue-specific, and functional validation will be required to resolve these issues.

## 5. Conclusions

THBS2 is a genetically prioritized and disease-associated matricellular molecule involved in extracellular matrix remodeling and specific chondrocyte states in knee osteoarthritis. Although MR analysis suggested an association between genetically predicted THBS2 expression and reduced KOA risk, this finding should be interpreted together with tissue-level transcriptomic evidence showing increased THBS2 expression in KOA cartilage. Single-cell analysis further revealed that THBS2 is mainly localized to fibrochondrocytes and osteogenic chondrocytes, suggesting its involvement in disease-associated cellular programs and cartilage microenvironment remodeling.

## Figures and Tables

**Figure 1:**
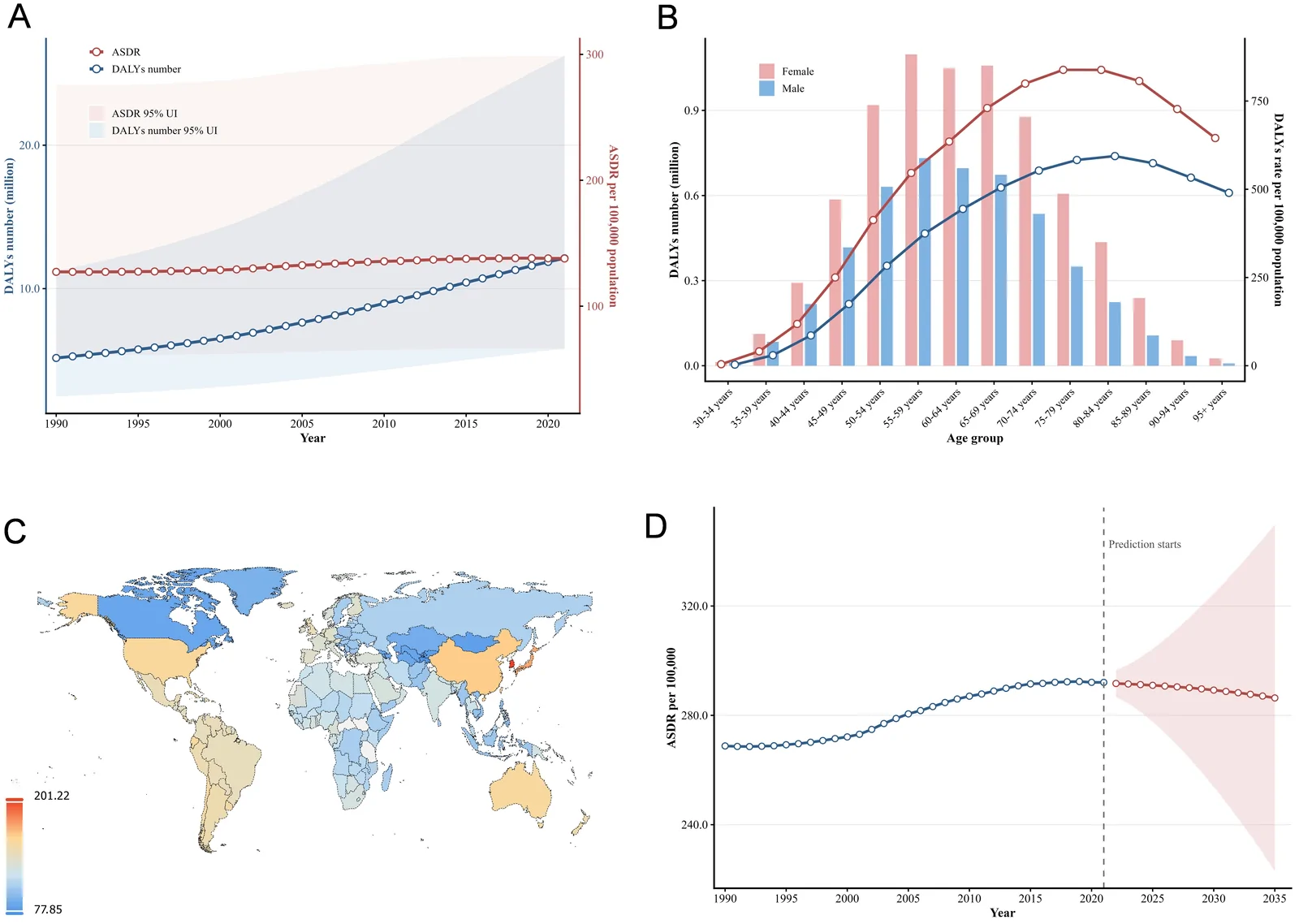
Global burden, demographic distribution, spatial heterogeneity, and projected trends of KOA based on GBD 2021. (**A**) Global trends in number of DALYs and ASDR of knee osteoarthritis from 1990 to 2021. (**B**) Age- and sex-specific distribution of knee osteoarthritis-related DALYs in 2021. Bars represent number of DALYs, and lines represent age-specific DALY rates. (**C**) Global spatial distribution of the age-standardized DALY rate of knee osteoarthritis across countries and territories in 2021. (**D**) Observed and projected global age-standardized DALY rate of knee osteoarthritis from 1990 to 2035 based on the BAPC model. DALYs, disability-adjusted life years; ASDR, age-standardized DALY rate; UI, uncertainty interval; KOA, knee osteoarthritis; GBD, Global Burden of Disease; BAPC, Bayesian age-period cohort.

**Figure 2:**
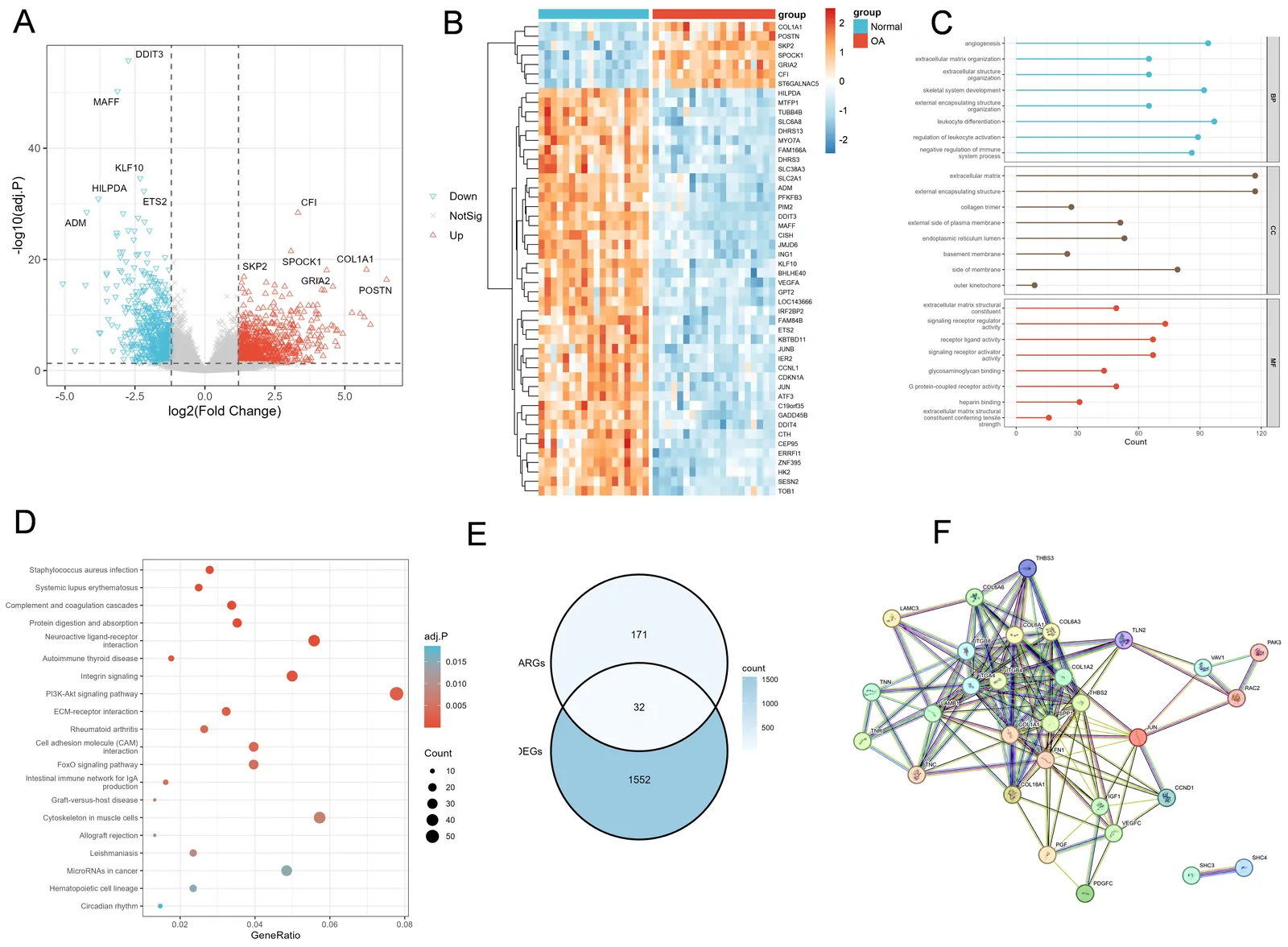
Identification and functional characterization of differentially expressed focal adhesion-related genes in KOA. (**A**) Volcano plot showing differentially expressed genes (DEGs) between KOA and control cartilage samples. (**B**) Heatmap showing the expression patterns of the top 50 DEGs across KOA and control samples. (**C**) Gene Ontology enrichment analysis of DEGs. The enriched biological processes were mainly associated with angiogenesis, extracellular matrix organization, extracellular structure organization, and skeletal system development. (**D**) KEGG pathway enrichment analysis of DEGs. (**E**) Venn diagram showing the intersection between DEGs and focal adhesion-related genes. (**F**) Protein–protein interaction network of differentially expressed focal adhesion-related genes. KOA, knee osteoarthritis; DEG, differentially expressed gene; GO, Gene Ontology; KEGG, Kyoto Encyclopedia of Genes and Genomes; ECM, extracellular matrix; PPI, protein-protein interaction.

**Figure 3:**
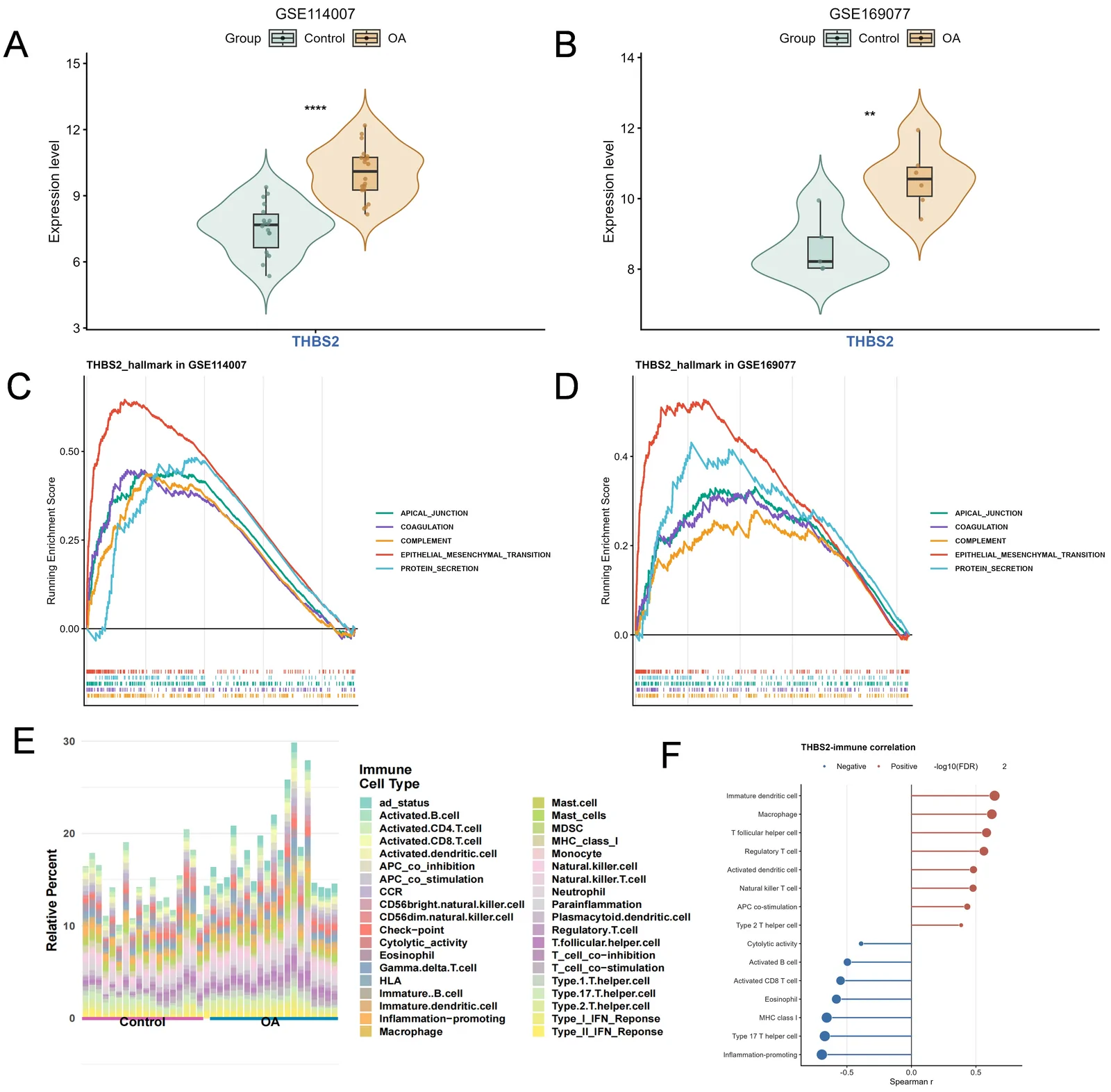
Expression validation, functional enrichment, and immune-related transcriptional signatures associated with THBS2 in KOA. (**A**) THBS2 expression in KOA and control cartilage samples from the discovery cohort GSE114007. (**B**) THBS2 expression in KOA and control cartilage samples from the validation cohort GSE169077. (**C**) Hallmark gene set enrichment analysis of THBS2-associated genes in GSE114007. (**D**) Hallmark gene set enrichment analysis of THBS2-associated genes in GSE169077. (**E**) Differences in immune-related ssGSEA signature scores between KOA and control cartilage samples. (**F**) Correlations between THBS2 expression and immune-related ssGSEA signature scores. These scores represent gene-set enrichment patterns and should not be interpreted as direct measurements of immune-cell abundance or proof of specific immune-cell populations in cartilage. KOA, knee osteoarthritis; THBS2, thrombospondin-2; GSEA, gene set enrichment analysis; ssGSEA, single-sample gene set enrichment analysis. ** *p* < 0.01; **** *p* < 0.0001.

**Figure 4:**
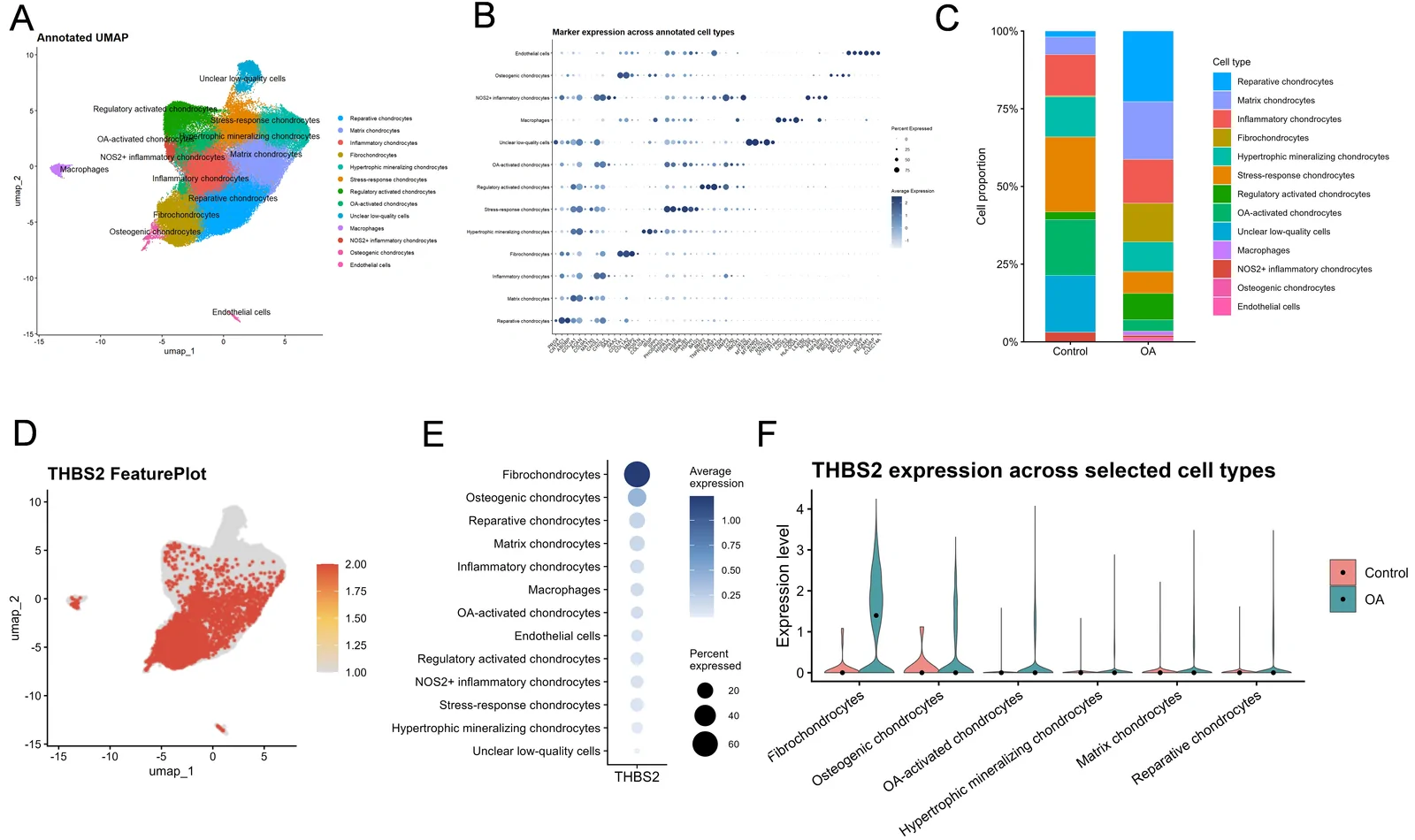
Single-cell transcriptomic atlas reveals predominant THBS2 expression in fibrochondrocytes and osteogenic chondrocytes in KOA cartilage. (**A**) UMAP visualization of annotated cell states or cell types in the single-cell RNA-seq dataset. (**B**) DotPlot showing the expression patterns of canonical marker genes across annotated cell populations. (**C**) Stacked bar plot showing pooled-cell relative proportions of annotated cell types in Control and OA groups. (**D**) FeaturePlot showing the distribution of THBS2 expression across the UMAP space. (**E**) DotPlot showing THBS2 expression across annotated cell populations. (**F**) Violin plot showing THBS2 expression across selected cell populations in Control and OA groups. KOA, knee osteoarthritis; UMAP, uniform manifold approximation and projection; RNA-seq, RNA sequencing.

**Figure 5:**
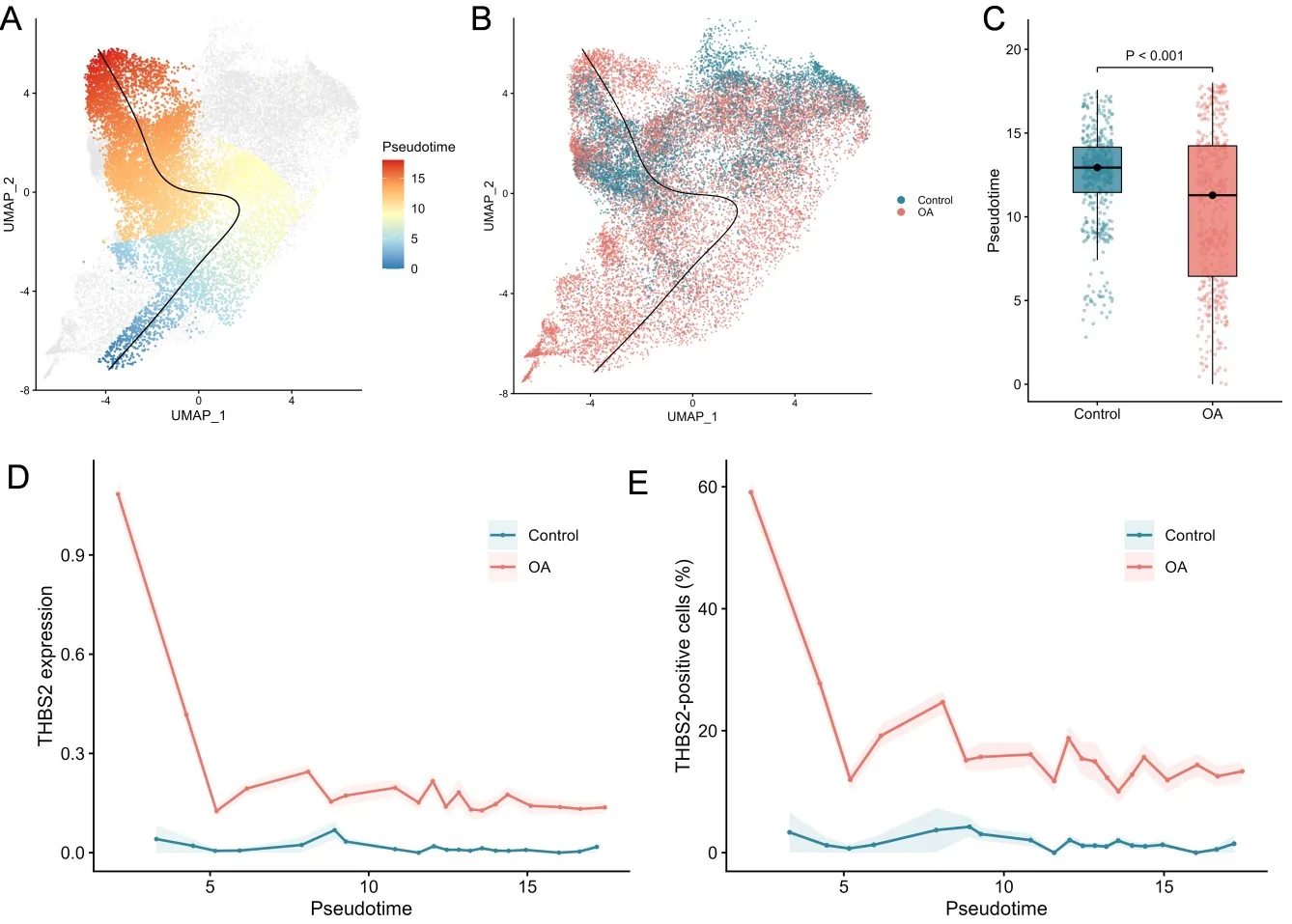
Distribution of THBS2-detectable cells along the jointly inferred pseudotime continuum. (**A**) UMAP visualization of the exploratory Slingshot-inferred continuum across chondrocyte-related populations; Control and OA cells were jointly used for trajectory inference. Cells are colored according to pseudotime, and the black curve indicates the inferred transcriptional continuum. (**B**) UMAP visualization of Control and OA chondrocytes within the shared state space. (**C**) Pooled-cell pseudotime distributions in Control and OA chondrocytes. No donor-level pseudotime test was performed. (**D**) Average THBS2 expression along the jointly inferred continuum in Control and OA chondrocytes. (**E**) Proportion of THBS2-detectable cells along the continuum. KOA, knee osteoarthritis; UMAP, uniform manifold approximation and projection.

**Figure 6:**
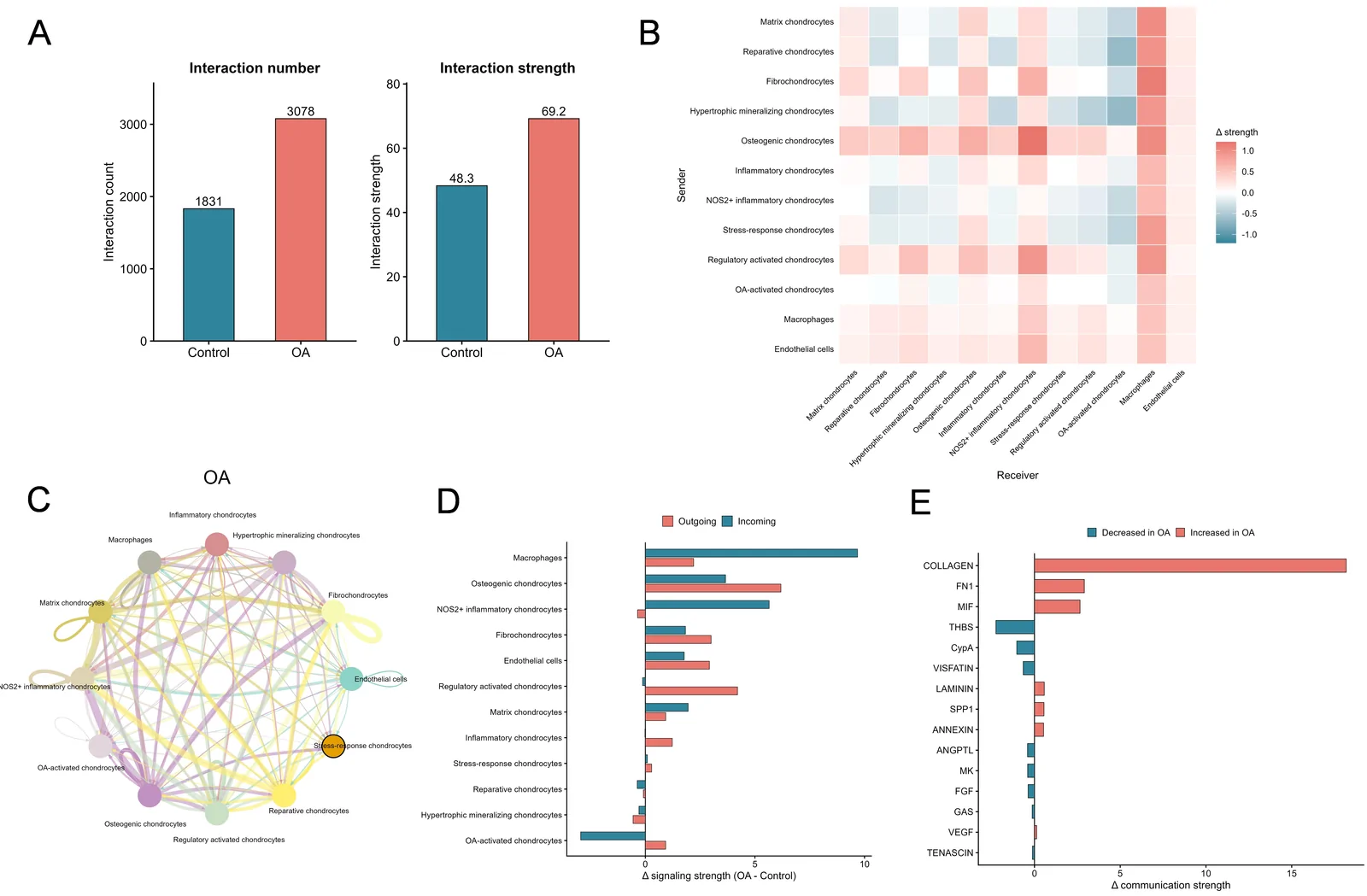
Exploratory CellChat comparison of pooled Control and OA cartilage networks. (**A**) Bar plots showing the total number and aggregate strength of inferred cell–cell interactions in pooled Control and pooled KOA networks. (**B**) Heatmap showing OA-minus-Control differences in inferred communication strength between sender and receiver populations. (**C**) Circular network plot showing the inferred intercellular communication network among annotated cell populations in the pooled KOA model. Edge thickness represents relative inferred communication strength. (**D**) Bar plot showing pooled-network differences in outgoing and incoming signaling strength across cell populations. (**E**) Pathway-level OA-minus-Control differences in inferred communication strength. KOA, knee osteoarthritis; ECM, extracellular matrix.

**Table 1:** Mendelian randomization analysis of the genetic associations between differentially expressed focal adhesion-related genes and knee osteoarthritis.

Gene	Primary MR Method	SNPs (*n*)	Beta	SE	OR (95% CI)	*p*	FDR-Adjusted *p*
THBS2	Inverse variance weighted	3	−0.332	0.118	0.72 (0.57–0.90)	0.0050	0.0422
SPP1	Inverse variance weighted	13	−0.085	0.039	0.92 (0.85–0.99)	0.0278	0.1573
JUN	Inverse variance weighted	8	0.163	0.081	1.18 (1.00–1.38)	0.0434	0.1831

## Data Availability

Publicly available datasets were analyzed in this study. Bulk transcriptomic data are available from the Gene Expression Omnibus under accession numbers GSE114007 and GSE169077, and single-cell RNA-sequencing data under GSE255460. The KOA GWAS data correspond to GCST005813 (EBI-a-GCST005813). GBD 2021 and eQTLGen summary statistics were obtained from their respective public resources. Additional results are included in the article and Supplementary Materials.

## Supplementary Materials

The following supporting information can be downloaded at https://www.mdpi.com/article/10.3390/genes17101299/s1.
