# Supplementary material for: THBS2 Marks Disease-Associated Chondrocyte States in Knee Osteoarthritis Cartilage: An Integrative Transcriptomic and Genetic Study
Source: Genes (Basel). 2026 Oct 18;17(10):1299. doi: 10.3390/genes17101299 (PMC13704032; doi:10.3390/genes17101299)
Supplement: Supplementary file 1 [file genes-17-01299-s001.zip › genes-4536589 figureS1-S6.pdf]

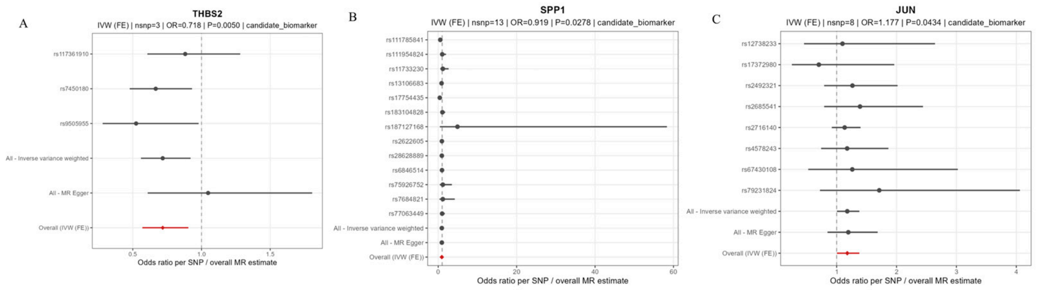

Supplementary Figure S1. Forest plots of Mendelian randomization estimates for selected focal adhesion–related candidate genes.

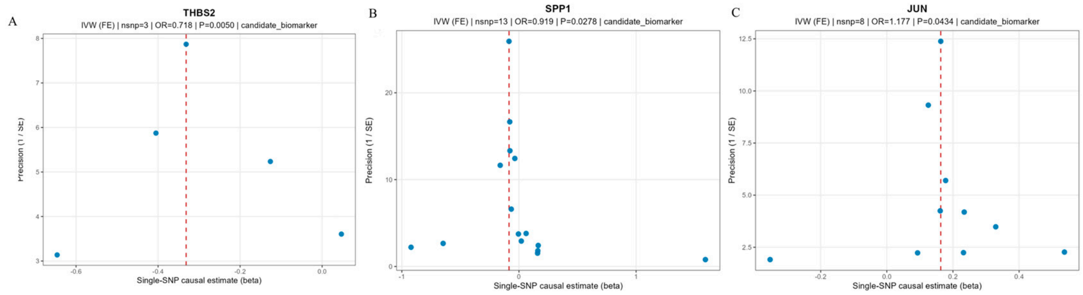

Supplementary Figure S2. Funnel plots for Mendelian randomization analysis of selected focal adhesion–related candidate genes.

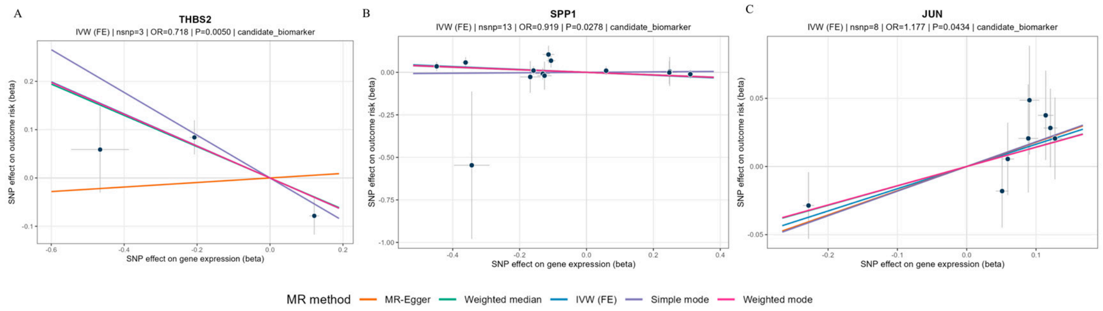

Supplementary Figure S3. Scatter plots of Mendelian randomization analysis for selected focal adhesion–related candidate genes.

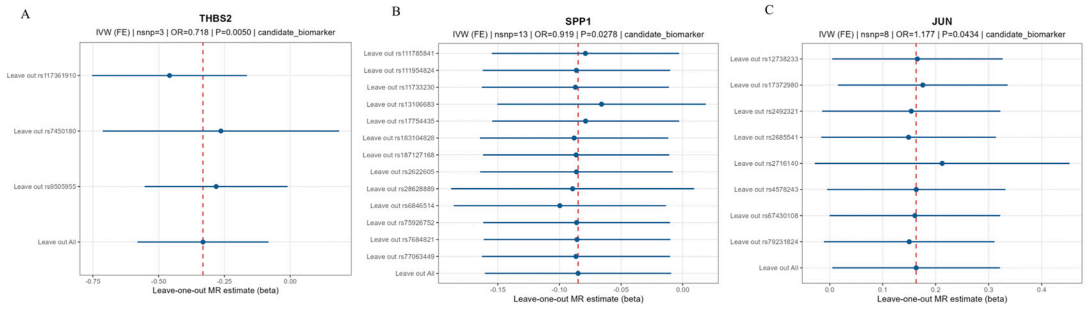

Supplementary Figure S4. Leave–one–out sensitivity analysis for selected focal adhesion–related candidate genes.

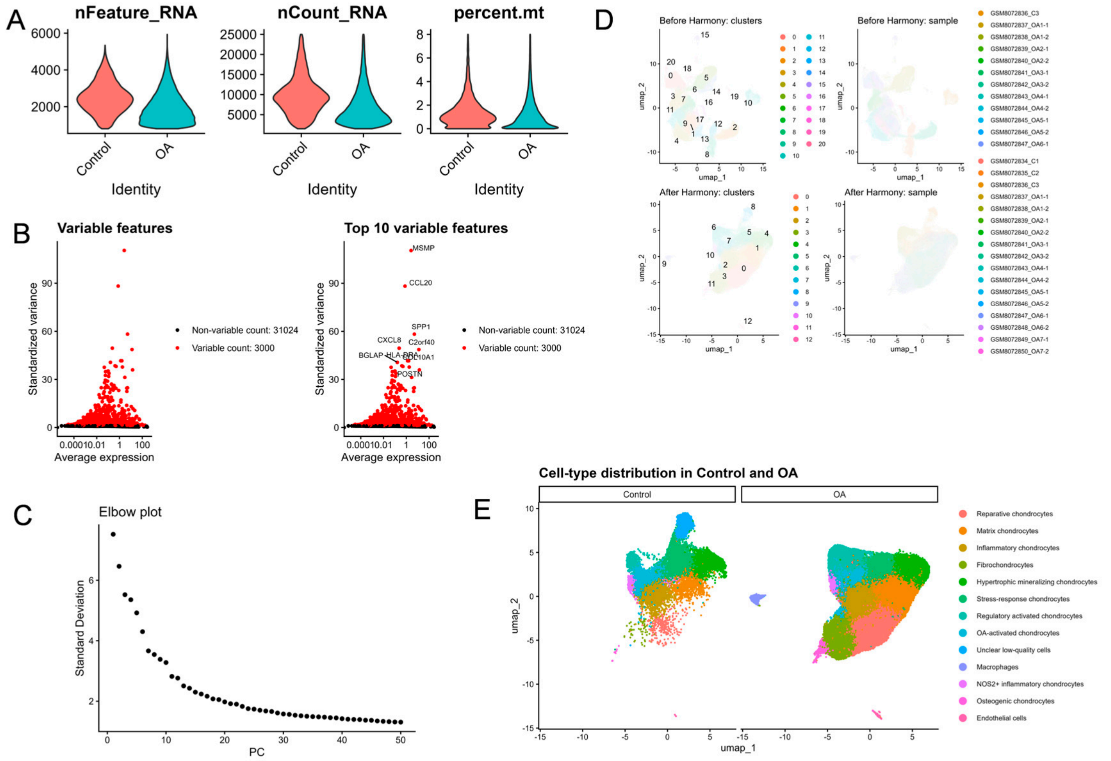

Supplementary Figure S5. Quality control, feature selection, dimensionality reduction, and integration of the single–cell RNA-seq dataset.

(A) Violin plots showing the distributions of nFeature_RNA, nCount_RNA, and mitochondrial gene percentage in Control and OA cells after quality control.(B) Identification of highly variable genes across the single–cell dataset, with the top 10 variable features labeled.(C) Elbow plot showing the standard deviation of principal components used to guide dimensionality reduction.(D) UMAP visualizations before and after Harmony integration, colored by unsupervised clusters and sample identity, demonstrating effective reduction of sample–specific batch effects.(E) UMAP visualization of annotated cell–type distributions in Control and KOA groups.RNA–seq, RNA sequencing; KOA, knee osteoarthritis; UMAP, uniform manifold approximation and projection; PC, principal component.

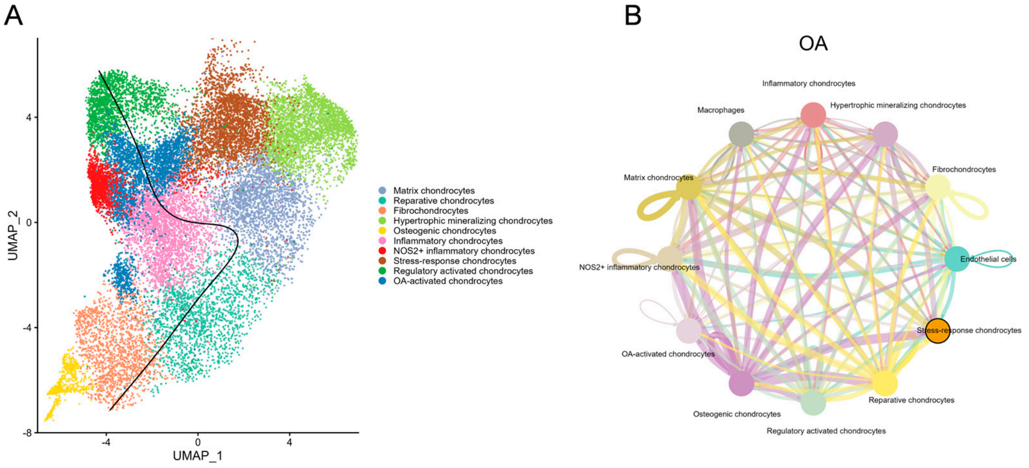

Supplementary Figure S6. Cell–type–resolved pseudotime trajectory and comparative interaction–number networks in KOA cartilage.

(A) UMAP visualization of chondrocyte–related cell populations overlaid with the Slingshot–inferred trajectory. Cells are colored according to annotated cell type, and the black curve indicates the inferred pseudotime trajectory.(B) Circular network plots showing CellChat–inferred interaction numbers among annotated cell populations in the Control and KOA groups. Edge thickness represents the relative number of inferred ligand–receptor interactions between cell populations. KOA, knee osteoarthritis; UMAP, uniform manifold approximation and projection.
